# Supplementary material for: Instruments used to assess gender-affirming healthcare access: A scoping review
Source: PLoS One. 2024 Jun 3;19(6):e0298821. doi: 10.1371/journal.pone.0298821 (PMC11146745; doi:10.1371/journal.pone.0298821)
Supplement: Attachment — Submitted filename: Response to Reviewers.docx [file pone.0298821.s003.docx]

23 Jan. 24

Dear Editor,

I hope this message finds you well. I am writing in response to the = feedback provided by the reviewers for our manuscript titled. I am grateful for the insightful comments and constructive suggestions that have been shared.

In the table below, I have addressed each of the reviewer's points and outlined the corresponding revisions made to enhance the clarity and quality of the manuscript. Your consideration of these revisions is highly appreciated. If you require anything further, then do let me know.

In addition, I attach the tracked changes and clean versions of the manuscript.

| Reviewer 1 comments: | Responses |
| --- | --- |
| Abstract/Introduction/Title | |
| 1. The background section could include a statement on the unique nature of the review and its implications (i.e. being the first of its kind) which is mentioned later in the Conclusion. | Paragraph added to the end of the introduction to address this point. |
| 2. Regarding choosing Levesque’s theory, could you mentioned others that were considered and why they did not fit with the review question/analysis approach? | A rationale for choosing Levesque’s theory was added to the theoretical underpinning section of the paper. |
| 3. Line 73 – remove “e.g.” from reference | Updated accordingly. |
| 4. Mention “university” librarian in the Abstract section | Updated accordingly. |
| 5. Transgender and non-binary representation in media is mentioned in Conclusion but not in background. I would suggest making a brief note about this in the Introduction to set the context. | Included a comment on media in the introduction. |
| 6. Suggestion that the scope of the review and implications is broader than the “instruments” mentioned in the title. Based on title, I expected article to focus strictly on evaluation/assessment tools. | I think in the introduction section the review outlines the breadth of the review in more detail here “A scoping review is the most appropriate type of review in this case because it allows for a comprehensive exploration of the existing literature, which is essential in identifying the range of healthcare access instruments used and the influencing factors on access in the transgender population.”. The title lists instruments as the overarching aim and the specific objectives detail the breadth again. Therefore, I have not opted to change this as I feel that it is appropriate. |
| Methods | |
| 1. Line 107 - Were the healthcare access experts based in Ireland or international? | Based in Ireland and updated. |
| 2. Line 136 – Were any further empirical articles found using Google Scholar as peer-reviewed articles are found this way too? | Yes, 8 studies were found this way and the process is described under ‘identification of relevant studies” and categorised as from websites in grey literature section. |
| 3. Line 146 – Use past tense i.e. “arose” | Updated to past tense. |
| 4. Line 146 – Was the criteria adapted after initial pilot? | Updated to include sentence ”No adaptations were required following the pilot”. |
| 5. State the third reviewer was also involved in full-text screening. | Updated |
| 6. Line 163 – What format were results shared with panel e.g. email, group discussion? | Updated sentence on same. |
| Results | |
| 1. Line 174 - PRISMA flowchart appears to be a screen grab from Word with spellchecked underlined words. Suggest included clean version. | Updated and made clean. |
| 2. Line 192 – I would include reference for this study. | Reference included as requested. |
| 3. The research question focuses on quantitative instruments. The results includes studies with PPI which is commonly a qualitative approach. Did these studies have quantitative elements? | Good point. Yes, the studies that were included had PPI but had quantitative approaches. I added a sentence for clarity on this point too. |
| Discussion | |
| 1. Suggestion of including separate paragraph that details the future policy, practice and research recommendations as a result of your review. Not required as also works with the threaded throughout Discussion. | I also feel that future research and recommendations are appropriately addressed threaded throughout the discussion and so no changes were made to this section. We discuss changes that HCP and policy makers could make. |

| Reviewer 2 comments: | Responses |
| --- | --- |
| Abstract/Introduction/Title | |
| The authors clearly identify their use of Levesque’s healthcare access theory; I encourage them to consider introducing the model more within their introduction and reason for selection. For example, they mention the model recognizes socioeconomic status, cultural beliefs, and health literacy. Some examples or further expansion on the relevance of these may be beneficial for the reader to apply to this population. | A detailed rationale for choosing Levesque was added to the theoretical underpinning section of the paper with reference to the points mentioned by Reviewer 2. |
| Methods | |
| Section 2.3 provides some details on the search and while the protocol is published elsewhere, it would be beneficial to provide some examples of search terms related to the concepts searched. | Included some examples for clarity. |
| I think it would be beneficial if the authors address the rationale for a grey literature search, why this was included and what this would add? | Included that it is a requirement for rigour in scoping reviews. |
| The authors mention sharing results with a pre-existing expert panel consisting of transgender and non-binary youths, I suggest the authors considering locating themselves within this work and analysis and how their own experiences or backgrounds may have contributed to shaping this work. I am also curious if the author’s could clarify why the group consisted of youths given this research does not have an age restriction or limitation specified. | Good suggestion. I have now included a section on our backgrounds and applied more clarity on the expert panel group. |
| Results | |
| Line 271 mentions “disparities observed between transfeminine and transmasculine individuals and between binary and non-binary healthcare seekers” but no mention of what these might include, some clarification here would be beneficial for a reader. | Added clarifying points on disparities with reference to non-binary vs binary, surgery and ethnicity and race and then linked to cultural and societal values. |
| Line 287 mentions “the present cohort of patients considered affordability as a crucial determinant for accessing healthcare services”, have the authors noted any difference here in terms of location and different healthcare coverage systems? Given the US is most highly representative I wonder if there were any differences to note. | Included a point on same noting the impact of waiting times and appropriateness of care in studies situated in Europe. |

Yours sincerely,

Seán Kearns

Author and project lead.
